# {μ-6,6′-Dimeth­oxy-2,2′-[butane-1,4-diylbis(nitrilo­methanylyl­idene)]diphenolato}trinitratocopper(II)samarium(III)

**DOI:** 10.1107/S1600536811031138

**Published:** 2011-08-06

**Authors:** Jing-Chun Xing, Bing Zhang, Wen-Zhi Li, Xiao-Guang Cui

**Affiliations:** a Department of Anesthesiology, the Second Affiliated Hospital, Harbin Medical University, Harbin 150081, People’s Republic of China

## Abstract

In the monomeric dinuclear title complex, [CuSm(C_20_H_22_N_2_O_4_)(NO_3_)_3_], the four-coordinate Cu^II^ ion has a square-planar geometry involving two O atoms and two N atoms of the deprotonated Schiff base ligand. The Sm^III^ ion is ten-coordinate, chelated by four O donor atoms of the Schiff base and two O atoms each from three bidentate nitrate groups, one of which is disordered over two sites in a 0.55 (7):0.45 (7) ratio.

## Related literature

For copper–lanthanide complexes of the same or similar Schiff bases, see: Xing *et al.* (2009 ▶, 2010 ▶).
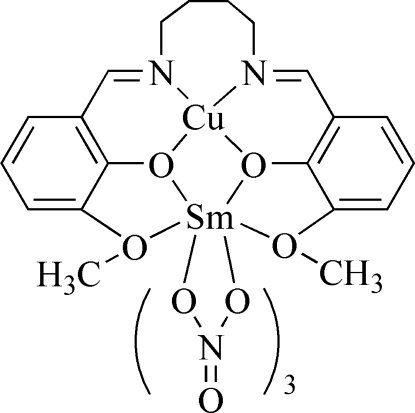


## Experimental

### 

#### Crystal data

[CuSm(C_20_H_22_N_2_O_4_)(NO_3_)_3_]
*M* *_r_* = 754.32
Monoclinic, 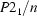

*a* = 11.764 (2) Å
*b* = 14.786 (3) Å
*c* = 15.004 (3) Å
β = 100.73 (3)°
*V* = 2564.3 (9) Å^3^
*Z* = 4
Mo *K*α radiation
μ = 3.17 mm^−1^
*T* = 295 K
0.24 × 0.18 × 0.15 mm

#### Data collection

Rigaku R-AXIS RAPID CCD diffractometer
Absorption correction: multi-scan (*ABSCOR*; Higashi, 1995 ▶) *T*_min_ = 0.516, *T*_max_ = 0.648
24339 measured reflections
5855 independent reflections
5046 reflections with *I* > 2σ(*I*)
*R*_int_ = 0.037

#### Refinement

*R*[*F* ^2^ > 2σ(*F* ^2^)] = 0.026
*wR*(*F* ^2^) = 0.062
*S* = 1.04
5855 reflections
373 parameters
H-atom parameters constrained
Δρ_max_ = 0.77 e Å^−3^
Δρ_min_ = −0.38 e Å^−3^

### 

Data collection: *RAPID-AUTO* (Rigaku, 1998 ▶); cell refinement: *RAPID-AUTO*; data reduction: *CrystalStructure* (Rigaku/MSC, 2002 ▶); program(s) used to solve structure: *SHELXS97* (Sheldrick, 2008 ▶); program(s) used to refine structure: *SHELXL97* (Sheldrick, 2008 ▶); molecular graphics: *SHELXTL* (Sheldrick, 2008 ▶); software used to prepare material for publication: *SHELXTL*.

## Supplementary Material

Crystal structure: contains datablock(s) I, global. DOI: 10.1107/S1600536811031138/zs2129sup1.cif

Structure factors: contains datablock(s) I. DOI: 10.1107/S1600536811031138/zs2129Isup2.hkl

Additional supplementary materials:  crystallographic information; 3D view; checkCIF report

## Figures and Tables

**Fig. 1.**
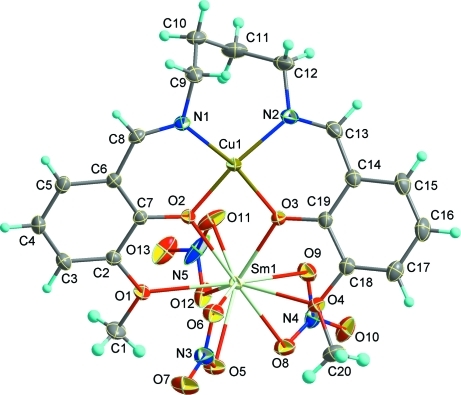
The molecular structure and atom numbering scheme for the title complex, showing 40% probability displacement ellipsoids. All H atoms have been omitted for clarity.

## supplementary crystallographic information

### Comment

In the monomeric dinuclear title complex, [CuSm(C_20_H_22_N_2_O_4_)(NO_3_)_3_], (Fig. 1) the four-coordinate Cu^II^ ion has square-planar geometry involving two O atoms and two N atoms of the deprotonated Schiff base. The Sm^III^ ion is ten-coordinate, chelated by four O donor atoms of the Schiff base and three bidentate nitrato groups, one of which is disordered over two sites (S.O.F. 0.55/0.45). This complex is isomorphous with the Cu^II^–Nd^III^ complex with the same ligand (Xing *et al.*, 2010) and has similar features to those reported for the Cu^II^–Lu^III^ complex with a similar ligand (Xing *et al.*, 2009)

### Experimental

The title complex was obtained by the reaction of copper(II) acetate monohydrate (0.0499 g, 0.25 mmol) with the Schiff base (0.1595 g, 0.25 mmol) in methanol/acetone (20 ml:5 ml) at room temperature. The mixture was then refluxed for 3 h after the addition of samarium(III) nitrate hexahydrate (0.1143 g, 0.25 mmol). The reaction mixture was cooled and filtered and diethyl ether was allowed to diffuse slowly into the solution. Single crystals were obtained after several days. Analysis: calculated for C_20_H_22_CuN_5_O_13_Sm: C, 31.82; H, 2.92; Cu, 8.42; N, 9.28; Sm, 19.93%. Found: C, 32.80; H, 2.94; Cu, 8.45; N, 9.29; Sm, 19.91%.

### Refinement

H atoms bound to C were placed in calculated positions and treated as riding on their parent atoms, with C—H = 0.93 Å (aromatic), C—H = 0.97 Å (methylene), and with *U*_iso_(H) = 1.2*U*_eq_(C) or C—H = 0.96 Å (methyl) and with *U*_iso_(H) = 1.5*U*_eq_(C). One of the nitrate groups incorporating N5 is disordered over two sites, with occupancies of 0.55 (7) and 0.45 (7).

### Crystal data

**Table:**

[CuSm(C_20_H_22_N_2_O_4_)(NO_3_)_3_]	*F*(000) = 1488
*M**_r_* = 754.32	*D*_x_ = 1.954 Mg m^−^^3^
Monoclinic, *P*2_1_/*n*	Mo *K*α radiation, λ = 0.71073 Å
Hall symbol: -P 2yn	Cell parameters from 19348 reflections
*a* = 11.764 (2) Å	θ = 3.1–27.4°
*b* = 14.786 (3) Å	µ = 3.17 mm^−^^1^
*c* = 15.004 (3) Å	*T* = 295 K
β = 100.73 (3)°	Prism, brown
*V* = 2564.3 (9) Å^3^	0.24 × 0.18 × 0.15 mm
*Z* = 4	

### Data collection

**Table:**

Rigaku R-AXIS RAPID CCD diffractometer	5855 independent reflections
Radiation source: fine-focus sealed tube	5046 reflections with *I* > 2σ(*I*)
graphite	*R*_int_ = 0.037
Detector resolution: 10.00 pixels mm^-1^	θ_max_ = 27.5°, θ_min_ = 3.1°
ω scans	*h* = −13→15
Absorption correction: multi-scan (*ABSCOR*; Higashi, 1995)	*k* = −19→19
*T*_min_ = 0.516, *T*_max_ = 0.648	*l* = −19→19
24339 measured reflections	

### Refinement

**Table:**

Refinement on *F*^2^	Primary atom site location: structure-invariant direct methods
Least-squares matrix: full	Secondary atom site location: difference Fourier map
*R*[*F*^2^ > 2σ(*F*^2^)] = 0.026	Hydrogen site location: inferred from neighbouring sites
*wR*(*F*^2^) = 0.062	H-atom parameters constrained
*S* = 1.04	*w* = 1/[σ^2^(*F*_o_^2^) + (0.0242*P*)^2^ + 1.6986*P*] where *P* = (*F*_o_^2^ + 2*F*_c_^2^)/3
5855 reflections	(Δ/σ)_max_ = 0.001
373 parameters	Δρ_max_ = 0.77 e Å^−^^3^
0 restraints	Δρ_min_ = −0.38 e Å^−^^3^

### Special details

**Table:**

Geometry. All e.s.d.'s (except the e.s.d. in the dihedral angle between two l.s. planes) are estimated using the full covariance matrix. The cell e.s.d.'s are taken into account individually in the estimation of e.s.d.'s in distances, angles and torsion angles; correlations between e.s.d.'s in cell parameters are only used when they are defined by crystal symmetry. An approximate (isotropic) treatment of cell e.s.d.'s is used for estimating e.s.d.'s involving l.s. planes.
Refinement. Refinement of *F*^2^ against ALL reflections. The weighted *R*-factor *wR* and goodness of fit *S* are based on *F*^2^, conventional *R*-factors *R* are based on *F*, with *F* set to zero for negative *F*^2^. The threshold expression of *F*^2^ > σ(*F*^2^) is used only for calculating *R*-factors(gt) *etc*. and is not relevant to the choice of reflections for refinement. *R*-factors based on *F*^2^ are statistically about twice as large as those based on *F*, and *R*- factors based on ALL data will be even larger.

### Fractional atomic coordinates and isotropic or equivalent isotropic displacement parameters (Å^2^)

**Table:**

	*x*	*y*	*z*	*U*_iso_*/*U*_eq_	Occ. (<1)
Sm1	0.259064 (11)	0.250880 (8)	0.565440 (9)	0.03052 (5)	
Cu1	0.54988 (3)	0.26907 (2)	0.53542 (2)	0.03185 (8)	
O1	0.25817 (15)	0.40859 (13)	0.64009 (13)	0.0407 (4)	
O2	0.43419 (14)	0.34352 (11)	0.57697 (12)	0.0339 (4)	
O3	0.41124 (15)	0.19822 (12)	0.49482 (13)	0.0384 (4)	
O4	0.20146 (16)	0.16351 (14)	0.41214 (13)	0.0439 (5)	
O5	0.07423 (16)	0.32911 (14)	0.50534 (14)	0.0471 (5)	
O6	0.21496 (18)	0.36420 (14)	0.43857 (14)	0.0495 (5)	
O7	0.0562 (2)	0.4400 (2)	0.4088 (2)	0.0954 (10)	
O8	0.08550 (17)	0.14963 (15)	0.57011 (15)	0.0531 (5)	
O9	0.25342 (19)	0.08703 (14)	0.59909 (16)	0.0531 (5)	
O10	0.1007 (3)	0.00645 (17)	0.6032 (2)	0.0905 (9)	
O11	0.3947 (3)	0.2257 (2)	0.7125 (2)	0.0969 (11)	
O12	0.2166 (3)	0.23958 (16)	0.72203 (19)	0.0736 (8)	
O13	0.317 (4)	0.256 (3)	0.8441 (11)	0.101 (9)	0.45 (7)
O13'	0.367 (3)	0.206 (4)	0.8420 (12)	0.123 (12)	0.55 (7)
N1	0.66513 (18)	0.36617 (14)	0.55494 (14)	0.0327 (5)	
N2	0.64898 (19)	0.16474 (15)	0.51218 (16)	0.0395 (5)	
N3	0.1128 (2)	0.37923 (17)	0.44897 (18)	0.0485 (6)	
N4	0.1437 (2)	0.07931 (17)	0.59096 (17)	0.0505 (6)	
N5	0.3202 (5)	0.2317 (3)	0.7614 (2)	0.1070 (18)	
C1	0.1677 (3)	0.4401 (2)	0.6854 (2)	0.0593 (9)	
H1A	0.2000	0.4539	0.7475	0.089*	
H1B	0.1100	0.3938	0.6833	0.089*	
H1C	0.1330	0.4934	0.6557	0.089*	
C2	0.3512 (2)	0.46618 (17)	0.64170 (16)	0.0320 (5)	
C3	0.3556 (2)	0.55389 (18)	0.67252 (18)	0.0402 (6)	
H3A	0.2931	0.5781	0.6943	0.048*	
C4	0.4534 (3)	0.60651 (18)	0.67108 (19)	0.0423 (6)	
H4A	0.4555	0.6662	0.6908	0.051*	
C5	0.5459 (2)	0.57090 (17)	0.64095 (18)	0.0374 (6)	
H5A	0.6112	0.6062	0.6409	0.045*	
C6	0.5435 (2)	0.48049 (16)	0.60967 (16)	0.0299 (5)	
C7	0.4445 (2)	0.42749 (16)	0.60824 (15)	0.0279 (5)	
C8	0.6461 (2)	0.44643 (17)	0.58155 (16)	0.0325 (5)	
H8A	0.7061	0.4877	0.5828	0.039*	
C9	0.7817 (2)	0.3491 (2)	0.5359 (2)	0.0442 (7)	
H9A	0.8172	0.4060	0.5239	0.053*	
H9B	0.7761	0.3113	0.4824	0.053*	
C10	0.8564 (2)	0.3025 (2)	0.6165 (2)	0.0538 (8)	
H10A	0.9332	0.2930	0.6033	0.065*	
H10B	0.8638	0.3418	0.6690	0.065*	
C11	0.8077 (3)	0.2122 (3)	0.6393 (2)	0.0570 (8)	
H11A	0.8654	0.1810	0.6833	0.068*	
H11B	0.7408	0.2228	0.6672	0.068*	
C12	0.7726 (2)	0.1522 (2)	0.5577 (2)	0.0531 (8)	
H12A	0.8222	0.1648	0.5143	0.064*	
H12B	0.7844	0.0896	0.5763	0.064*	
C13	0.6102 (2)	0.10028 (19)	0.4572 (2)	0.0447 (7)	
H13A	0.6641	0.0572	0.4473	0.054*	
C14	0.4936 (2)	0.08675 (18)	0.40927 (19)	0.0391 (6)	
C15	0.4736 (3)	0.0181 (2)	0.3426 (2)	0.0494 (7)	
H15A	0.5354	−0.0152	0.3293	0.059*	
C16	0.3637 (3)	0.0005 (2)	0.2974 (2)	0.0539 (8)	
H16A	0.3516	−0.0439	0.2528	0.065*	
C17	0.2701 (3)	0.0484 (2)	0.31751 (19)	0.0481 (7)	
H17A	0.1958	0.0365	0.2860	0.058*	
C18	0.2879 (2)	0.11355 (18)	0.38434 (17)	0.0371 (6)	
C19	0.3997 (2)	0.13440 (17)	0.43070 (17)	0.0340 (5)	
C20	0.0871 (3)	0.1558 (3)	0.3594 (3)	0.0689 (10)	
H20A	0.0881	0.1725	0.2978	0.103*	
H20B	0.0358	0.1952	0.3839	0.103*	
H20C	0.0609	0.0944	0.3613	0.103*	

### Atomic displacement parameters (Å^2^)

**Table:**

	*U*^11^	*U*^22^	*U*^33^	*U*^12^	*U*^13^	*U*^23^
Sm1	0.02418 (8)	0.03020 (8)	0.03794 (8)	−0.00118 (5)	0.00772 (5)	0.00061 (5)
Cu1	0.02343 (15)	0.03144 (15)	0.04145 (17)	−0.00075 (12)	0.00804 (12)	−0.00423 (13)
O1	0.0304 (10)	0.0415 (10)	0.0548 (11)	−0.0022 (8)	0.0197 (8)	−0.0107 (9)
O2	0.0270 (9)	0.0300 (9)	0.0474 (10)	−0.0026 (7)	0.0136 (8)	−0.0075 (8)
O3	0.0270 (9)	0.0374 (10)	0.0518 (11)	−0.0039 (8)	0.0099 (8)	−0.0142 (8)
O4	0.0307 (10)	0.0502 (12)	0.0477 (11)	−0.0013 (9)	−0.0005 (8)	−0.0100 (9)
O5	0.0328 (10)	0.0522 (12)	0.0572 (12)	0.0006 (9)	0.0102 (9)	0.0127 (10)
O6	0.0443 (12)	0.0477 (12)	0.0592 (13)	−0.0025 (10)	0.0168 (10)	0.0125 (10)
O7	0.0728 (18)	0.084 (2)	0.127 (2)	0.0257 (15)	0.0114 (17)	0.0594 (19)
O8	0.0372 (11)	0.0511 (13)	0.0710 (14)	−0.0051 (10)	0.0099 (10)	0.0080 (11)
O9	0.0499 (13)	0.0390 (11)	0.0714 (14)	0.0056 (9)	0.0141 (11)	0.0060 (10)
O10	0.111 (2)	0.0542 (15)	0.104 (2)	−0.0436 (16)	0.0121 (18)	0.0118 (15)
O11	0.076 (2)	0.134 (3)	0.0671 (18)	−0.0499 (19)	−0.0231 (16)	0.0412 (18)
O12	0.106 (3)	0.0643 (16)	0.0575 (15)	−0.0322 (15)	0.0338 (16)	−0.0022 (12)
O13	0.139 (13)	0.130 (16)	0.032 (4)	−0.053 (14)	0.006 (5)	0.012 (5)
O13'	0.129 (13)	0.17 (2)	0.052 (4)	−0.086 (15)	−0.024 (6)	0.047 (8)
N1	0.0280 (11)	0.0337 (11)	0.0384 (11)	−0.0037 (9)	0.0110 (9)	−0.0010 (9)
N2	0.0279 (11)	0.0384 (12)	0.0541 (14)	0.0034 (9)	0.0123 (10)	0.0035 (11)
N3	0.0422 (14)	0.0422 (13)	0.0590 (16)	0.0010 (11)	0.0038 (12)	0.0112 (12)
N4	0.0619 (17)	0.0402 (14)	0.0485 (14)	−0.0158 (13)	0.0080 (12)	0.0000 (11)
N5	0.139 (4)	0.128 (3)	0.0450 (18)	−0.100 (3)	−0.005 (2)	0.018 (2)
C1	0.0386 (17)	0.066 (2)	0.081 (2)	−0.0029 (15)	0.0313 (16)	−0.0225 (18)
C2	0.0305 (13)	0.0332 (13)	0.0331 (12)	−0.0017 (10)	0.0082 (10)	−0.0022 (10)
C3	0.0394 (15)	0.0387 (14)	0.0439 (15)	0.0068 (12)	0.0115 (12)	−0.0079 (12)
C4	0.0455 (16)	0.0306 (13)	0.0506 (16)	−0.0007 (12)	0.0085 (13)	−0.0102 (12)
C5	0.0396 (15)	0.0316 (13)	0.0395 (14)	−0.0057 (11)	0.0038 (11)	−0.0040 (11)
C6	0.0293 (12)	0.0305 (12)	0.0293 (12)	−0.0020 (10)	0.0043 (10)	0.0003 (10)
C7	0.0295 (12)	0.0279 (11)	0.0258 (11)	0.0006 (10)	0.0039 (9)	−0.0005 (9)
C8	0.0276 (12)	0.0353 (13)	0.0347 (13)	−0.0068 (10)	0.0065 (10)	0.0017 (11)
C9	0.0352 (15)	0.0410 (15)	0.0624 (18)	−0.0061 (12)	0.0248 (13)	−0.0020 (13)
C10	0.0262 (14)	0.067 (2)	0.068 (2)	−0.0005 (14)	0.0084 (14)	−0.0153 (17)
C11	0.0349 (16)	0.079 (2)	0.0551 (19)	0.0086 (16)	0.0026 (14)	0.0160 (18)
C12	0.0307 (15)	0.0438 (17)	0.083 (2)	0.0058 (12)	0.0060 (15)	0.0105 (16)
C13	0.0401 (15)	0.0364 (14)	0.0628 (19)	0.0057 (12)	0.0230 (14)	−0.0036 (13)
C14	0.0412 (15)	0.0339 (13)	0.0456 (15)	−0.0029 (11)	0.0170 (12)	−0.0035 (11)
C15	0.062 (2)	0.0406 (15)	0.0518 (17)	−0.0009 (14)	0.0264 (15)	−0.0087 (13)
C16	0.072 (2)	0.0494 (18)	0.0438 (16)	−0.0061 (16)	0.0190 (15)	−0.0171 (14)
C17	0.0563 (18)	0.0505 (17)	0.0362 (14)	−0.0104 (14)	0.0058 (13)	−0.0070 (13)
C18	0.0398 (15)	0.0363 (14)	0.0357 (13)	−0.0030 (11)	0.0087 (11)	−0.0018 (11)
C19	0.0381 (14)	0.0294 (12)	0.0360 (13)	−0.0033 (11)	0.0105 (11)	−0.0039 (10)
C20	0.0398 (18)	0.085 (3)	0.073 (2)	0.0021 (17)	−0.0112 (16)	−0.019 (2)

### Geometric parameters (Å, °)

**Table:**

Sm1—O3	2.3748 (18)	C1—H1C	0.9600
Sm1—O2	2.4532 (17)	C2—C3	1.375 (3)
Sm1—O9	2.478 (2)	C2—C7	1.410 (3)
Sm1—O5	2.480 (2)	C3—C4	1.393 (4)
Sm1—O12	2.497 (3)	C3—H3A	0.9300
Sm1—O11	2.499 (3)	C4—C5	1.359 (4)
Sm1—O6	2.516 (2)	C4—H4A	0.9300
Sm1—O8	2.543 (2)	C5—C6	1.415 (3)
Sm1—O1	2.5879 (19)	C5—H5A	0.9300
Sm1—O4	2.6151 (19)	C6—C7	1.401 (3)
Sm1—N5	2.908 (3)	C6—C8	1.442 (3)
Sm1—N3	2.915 (2)	C8—H8A	0.9300
Cu1—O3	1.9388 (18)	C9—C10	1.521 (4)
Cu1—O2	1.9412 (17)	C9—H9A	0.9700
Cu1—N1	1.959 (2)	C9—H9B	0.9700
Cu1—N2	2.003 (2)	C10—C11	1.517 (5)
O1—C2	1.384 (3)	C10—H10A	0.9700
O1—C1	1.443 (3)	C10—H10B	0.9700
O2—C7	1.325 (3)	C11—C12	1.506 (5)
O3—C19	1.337 (3)	C11—H11A	0.9700
O4—C18	1.382 (3)	C11—H11B	0.9700
O4—C20	1.432 (3)	C12—H12A	0.9700
O5—N3	1.270 (3)	C12—H12B	0.9700
O6—N3	1.260 (3)	C13—C14	1.439 (4)
O7—N3	1.210 (3)	C13—H13A	0.9300
O8—N4	1.252 (3)	C14—C19	1.397 (4)
O9—N4	1.278 (3)	C14—C15	1.413 (4)
O10—N4	1.219 (3)	C15—C16	1.368 (4)
O11—N5	1.246 (6)	C15—H15A	0.9300
O12—N5	1.258 (6)	C16—C17	1.388 (4)
O13—N5	1.298 (14)	C16—H16A	0.9300
O13'—N5	1.292 (15)	C17—C18	1.378 (4)
N1—C8	1.285 (3)	C17—H17A	0.9300
N1—C9	1.474 (3)	C18—C19	1.404 (4)
N2—C13	1.287 (4)	C20—H20A	0.9600
N2—C12	1.498 (3)	C20—H20B	0.9600
C1—H1A	0.9600	C20—H20C	0.9600
C1—H1B	0.9600		
			
O3—Sm1—O2	61.42 (6)	O7—N3—O6	121.2 (3)
O3—Sm1—O9	79.71 (7)	O7—N3—O5	122.2 (3)
O2—Sm1—O9	125.84 (7)	O6—N3—O5	116.5 (2)
O3—Sm1—O5	132.34 (7)	O7—N3—Sm1	172.1 (2)
O2—Sm1—O5	115.67 (6)	O6—N3—Sm1	59.28 (13)
O9—Sm1—O5	118.37 (7)	O5—N3—Sm1	57.66 (13)
O3—Sm1—O12	134.22 (10)	O10—N4—O8	123.3 (3)
O2—Sm1—O12	106.48 (8)	O10—N4—O9	120.3 (3)
O9—Sm1—O12	74.05 (8)	O8—N4—O9	116.4 (2)
O5—Sm1—O12	93.31 (10)	O10—N4—Sm1	177.0 (3)
O3—Sm1—O11	86.26 (11)	O8—N4—Sm1	59.64 (13)
O2—Sm1—O11	68.35 (8)	O9—N4—Sm1	56.80 (13)
O9—Sm1—O11	73.23 (9)	O11—N5—O12	117.1 (3)
O5—Sm1—O11	139.74 (11)	O11—N5—O13'	108 (2)
O12—Sm1—O11	50.64 (12)	O12—N5—O13'	132.7 (16)
O3—Sm1—O6	86.98 (7)	O11—N5—O13	136.8 (15)
O2—Sm1—O6	74.56 (7)	O12—N5—O13	103 (2)
O9—Sm1—O6	142.70 (7)	O11—N5—Sm1	58.69 (18)
O5—Sm1—O6	51.02 (7)	O12—N5—Sm1	58.66 (18)
O12—Sm1—O6	135.00 (9)	O13'—N5—Sm1	163 (3)
O11—Sm1—O6	140.81 (8)	O13—N5—Sm1	153 (3)
O3—Sm1—O8	119.67 (7)	O1—C1—H1A	109.5
O2—Sm1—O8	174.11 (6)	O1—C1—H1B	109.5
O9—Sm1—O8	50.72 (7)	H1A—C1—H1B	109.5
O5—Sm1—O8	68.32 (7)	O1—C1—H1C	109.5
O12—Sm1—O8	68.43 (9)	H1A—C1—H1C	109.5
O11—Sm1—O8	105.78 (9)	H1B—C1—H1C	109.5
O6—Sm1—O8	111.05 (7)	C3—C2—O1	124.5 (2)
O3—Sm1—O1	123.55 (6)	C3—C2—C7	121.3 (2)
O2—Sm1—O1	62.44 (6)	O1—C2—C7	114.2 (2)
O9—Sm1—O1	142.22 (7)	C2—C3—C4	120.1 (3)
O5—Sm1—O1	70.31 (7)	C2—C3—H3A	120.0
O12—Sm1—O1	68.62 (7)	C4—C3—H3A	120.0
O11—Sm1—O1	78.77 (10)	C5—C4—C3	120.2 (2)
O6—Sm1—O1	73.17 (7)	C5—C4—H4A	119.9
O8—Sm1—O1	116.78 (7)	C3—C4—H4A	119.9
O3—Sm1—O4	62.55 (6)	C4—C5—C6	120.6 (3)
O2—Sm1—O4	114.50 (6)	C4—C5—H5A	119.7
O9—Sm1—O4	71.57 (7)	C6—C5—H5A	119.7
O5—Sm1—O4	81.05 (7)	C7—C6—C5	119.9 (2)
O12—Sm1—O4	136.89 (8)	C7—C6—C8	122.6 (2)
O11—Sm1—O4	136.19 (10)	C5—C6—C8	117.4 (2)
O6—Sm1—O4	71.37 (7)	O2—C7—C6	123.4 (2)
O8—Sm1—O4	69.85 (7)	O2—C7—C2	118.7 (2)
O1—Sm1—O4	143.57 (6)	C6—C7—C2	117.9 (2)
O3—Sm1—N5	110.73 (13)	N1—C8—C6	128.1 (2)
O2—Sm1—N5	86.37 (9)	N1—C8—H8A	115.9
O9—Sm1—N5	73.12 (10)	C6—C8—H8A	115.9
O5—Sm1—N5	116.63 (14)	N1—C9—C10	110.1 (2)
O12—Sm1—N5	25.48 (13)	N1—C9—H9A	109.6
O11—Sm1—N5	25.22 (14)	C10—C9—H9A	109.6
O6—Sm1—N5	143.82 (10)	N1—C9—H9B	109.6
O8—Sm1—N5	87.91 (9)	C10—C9—H9B	109.6
O1—Sm1—N5	70.76 (10)	H9A—C9—H9B	108.2
O4—Sm1—N5	144.69 (9)	C11—C10—C9	113.1 (2)
O3—Sm1—N3	110.85 (7)	C11—C10—H10A	109.0
O2—Sm1—N3	94.19 (7)	C9—C10—H10A	109.0
O9—Sm1—N3	136.33 (7)	C11—C10—H10B	109.0
O5—Sm1—N3	25.64 (7)	C9—C10—H10B	109.0
O12—Sm1—N3	114.10 (10)	H10A—C10—H10B	107.8
O11—Sm1—N3	146.62 (10)	C12—C11—C10	113.1 (3)
O6—Sm1—N3	25.50 (7)	C12—C11—H11A	109.0
O8—Sm1—N3	90.71 (7)	C10—C11—H11A	109.0
O1—Sm1—N3	67.87 (7)	C12—C11—H11B	109.0
O4—Sm1—N3	76.52 (7)	C10—C11—H11B	109.0
N5—Sm1—N3	132.61 (13)	H11A—C11—H11B	107.8
O3—Cu1—O2	78.94 (7)	N2—C12—C11	113.2 (3)
O3—Cu1—N1	164.02 (8)	N2—C12—H12A	108.9
O2—Cu1—N1	92.49 (8)	C11—C12—H12A	108.9
O3—Cu1—N2	90.69 (9)	N2—C12—H12B	108.9
O2—Cu1—N2	163.38 (8)	C11—C12—H12B	108.9
N1—Cu1—N2	100.45 (9)	H12A—C12—H12B	107.7
O3—Cu1—Sm1	39.03 (5)	N2—C13—C14	127.9 (3)
O2—Cu1—Sm1	41.49 (5)	N2—C13—H13A	116.0
N1—Cu1—Sm1	133.98 (6)	C14—C13—H13A	116.0
N2—Cu1—Sm1	124.80 (7)	C19—C14—C15	119.3 (3)
C2—O1—C1	116.4 (2)	C19—C14—C13	122.4 (2)
C2—O1—Sm1	119.68 (14)	C15—C14—C13	118.1 (3)
C1—O1—Sm1	123.88 (17)	C16—C15—C14	120.3 (3)
C7—O2—Cu1	128.45 (15)	C16—C15—H15A	119.8
C7—O2—Sm1	124.64 (14)	C14—C15—H15A	119.8
Cu1—O2—Sm1	106.89 (7)	C15—C16—C17	120.7 (3)
C19—O3—Cu1	125.05 (16)	C15—C16—H16A	119.7
C19—O3—Sm1	124.68 (15)	C17—C16—H16A	119.7
Cu1—O3—Sm1	110.04 (8)	C18—C17—C16	119.7 (3)
C18—O4—C20	117.3 (2)	C18—C17—H17A	120.2
C18—O4—Sm1	116.40 (15)	C16—C17—H17A	120.2
C20—O4—Sm1	126.02 (19)	C17—C18—O4	125.0 (3)
N3—O5—Sm1	96.70 (15)	C17—C18—C19	121.0 (3)
N3—O6—Sm1	95.21 (16)	O4—C18—C19	114.0 (2)
N4—O8—Sm1	95.22 (16)	O3—C19—C14	122.9 (2)
N4—O9—Sm1	97.63 (16)	O3—C19—C18	118.1 (2)
N5—O11—Sm1	96.1 (3)	C14—C19—C18	118.9 (2)
N5—O12—Sm1	95.9 (3)	O4—C20—H20A	109.5
C8—N1—C9	116.1 (2)	O4—C20—H20B	109.5
C8—N1—Cu1	124.59 (18)	H20A—C20—H20B	109.5
C9—N1—Cu1	119.26 (17)	O4—C20—H20C	109.5
C13—N2—C12	113.5 (2)	H20A—C20—H20C	109.5
C13—N2—Cu1	122.24 (19)	H20B—C20—H20C	109.5
C12—N2—Cu1	124.24 (19)		
			
O2—Sm1—Cu1—O3	−159.24 (12)	O5—Sm1—O11—N5	28.5 (3)
O9—Sm1—Cu1—O3	51.08 (11)	O12—Sm1—O11—N5	−3.0 (2)
O5—Sm1—Cu1—O3	−98.96 (12)	O6—Sm1—O11—N5	113.2 (2)
O12—Sm1—Cu1—O3	127.99 (11)	O8—Sm1—O11—N5	−46.4 (3)
O11—Sm1—Cu1—O3	119.13 (13)	O1—Sm1—O11—N5	68.5 (3)
O6—Sm1—Cu1—O3	−91.65 (11)	O4—Sm1—O11—N5	−123.6 (3)
O8—Sm1—Cu1—O3	31.90 (13)	N3—Sm1—O11—N5	70.4 (3)
O1—Sm1—Cu1—O3	−164.40 (10)	O3—Sm1—O12—N5	26.6 (3)
O4—Sm1—Cu1—O3	−20.46 (10)	O2—Sm1—O12—N5	−39.3 (3)
N5—Sm1—Cu1—O3	124.64 (13)	O9—Sm1—O12—N5	84.1 (2)
N3—Sm1—Cu1—O3	−96.58 (11)	O5—Sm1—O12—N5	−157.3 (2)
O3—Sm1—Cu1—O2	159.24 (12)	O11—Sm1—O12—N5	3.0 (2)
O9—Sm1—Cu1—O2	−149.68 (10)	O6—Sm1—O12—N5	−123.7 (2)
O5—Sm1—Cu1—O2	60.28 (11)	O8—Sm1—O12—N5	137.6 (3)
O12—Sm1—Cu1—O2	−72.77 (10)	O1—Sm1—O12—N5	−89.9 (2)
O11—Sm1—Cu1—O2	−81.64 (12)	O4—Sm1—O12—N5	122.3 (2)
O6—Sm1—Cu1—O2	67.59 (10)	N3—Sm1—O12—N5	−141.7 (2)
O8—Sm1—Cu1—O2	−168.86 (12)	O3—Cu1—N1—C8	53.8 (4)
O1—Sm1—Cu1—O2	−5.16 (9)	O2—Cu1—N1—C8	−3.2 (2)
O4—Sm1—Cu1—O2	138.78 (9)	N2—Cu1—N1—C8	−172.7 (2)
N5—Sm1—Cu1—O2	−76.12 (12)	Sm1—Cu1—N1—C8	−2.8 (3)
N3—Sm1—Cu1—O2	62.66 (10)	O3—Cu1—N1—C9	−124.4 (3)
O3—Sm1—Cu1—N1	158.61 (13)	O2—Cu1—N1—C9	178.70 (19)
O2—Sm1—Cu1—N1	−0.63 (12)	N2—Cu1—N1—C9	9.2 (2)
O9—Sm1—Cu1—N1	−150.32 (10)	Sm1—Cu1—N1—C9	179.12 (15)
O5—Sm1—Cu1—N1	59.64 (11)	O3—Cu1—N2—C13	21.8 (2)
O12—Sm1—Cu1—N1	−73.40 (11)	O2—Cu1—N2—C13	72.8 (4)
O11—Sm1—Cu1—N1	−82.27 (13)	N1—Cu1—N2—C13	−146.6 (2)
O6—Sm1—Cu1—N1	66.96 (10)	Sm1—Cu1—N2—C13	42.2 (3)
O8—Sm1—Cu1—N1	−169.49 (13)	O3—Cu1—N2—C12	−156.5 (2)
O1—Sm1—Cu1—N1	−5.79 (10)	O2—Cu1—N2—C12	−105.5 (3)
O4—Sm1—Cu1—N1	138.14 (10)	N1—Cu1—N2—C12	35.0 (2)
N5—Sm1—Cu1—N1	−76.75 (13)	Sm1—Cu1—N2—C12	−136.2 (2)
N3—Sm1—Cu1—N1	62.03 (10)	Sm1—O6—N3—O7	170.8 (3)
O3—Sm1—Cu1—N2	−33.46 (12)	Sm1—O6—N3—O5	−7.2 (3)
O2—Sm1—Cu1—N2	167.30 (12)	Sm1—O5—N3—O7	−170.7 (3)
O9—Sm1—Cu1—N2	17.62 (10)	Sm1—O5—N3—O6	7.4 (3)
O5—Sm1—Cu1—N2	−132.42 (11)	O3—Sm1—N3—O6	−21.65 (18)
O12—Sm1—Cu1—N2	94.53 (11)	O2—Sm1—N3—O6	39.32 (17)
O11—Sm1—Cu1—N2	85.66 (12)	O9—Sm1—N3—O6	−118.66 (17)
O6—Sm1—Cu1—N2	−125.11 (10)	O5—Sm1—N3—O6	−172.3 (3)
O8—Sm1—Cu1—N2	−1.56 (13)	O12—Sm1—N3—O6	149.46 (16)
O1—Sm1—Cu1—N2	162.14 (9)	O11—Sm1—N3—O6	95.2 (2)
O4—Sm1—Cu1—N2	−53.93 (9)	O8—Sm1—N3—O6	−143.95 (17)
N5—Sm1—Cu1—N2	91.18 (12)	O1—Sm1—N3—O6	97.27 (17)
N3—Sm1—Cu1—N2	−130.04 (10)	O4—Sm1—N3—O6	−74.89 (17)
O3—Sm1—O1—C2	−1.4 (2)	N5—Sm1—N3—O6	128.24 (18)
O2—Sm1—O1—C2	5.10 (16)	O3—Sm1—N3—O5	150.69 (16)
O9—Sm1—O1—C2	118.97 (18)	O2—Sm1—N3—O5	−148.34 (16)
O5—Sm1—O1—C2	−129.79 (18)	O9—Sm1—N3—O5	53.7 (2)
O12—Sm1—O1—C2	128.3 (2)	O12—Sm1—N3—O5	−38.20 (18)
O11—Sm1—O1—C2	76.39 (18)	O11—Sm1—N3—O5	−92.5 (2)
O6—Sm1—O1—C2	−75.93 (18)	O6—Sm1—N3—O5	172.3 (3)
O8—Sm1—O1—C2	178.54 (16)	O8—Sm1—N3—O5	28.39 (17)
O4—Sm1—O1—C2	−89.52 (19)	O1—Sm1—N3—O5	−90.39 (17)
N5—Sm1—O1—C2	101.2 (2)	O4—Sm1—N3—O5	97.45 (17)
N3—Sm1—O1—C2	−102.43 (18)	N5—Sm1—N3—O5	−59.4 (2)
O3—Sm1—O1—C1	−178.1 (2)	Sm1—O8—N4—O10	179.3 (3)
O2—Sm1—O1—C1	−171.6 (2)	Sm1—O8—N4—O9	−0.7 (3)
O9—Sm1—O1—C1	−57.7 (3)	Sm1—O9—N4—O10	−179.3 (3)
O5—Sm1—O1—C1	53.5 (2)	Sm1—O9—N4—O8	0.8 (3)
O12—Sm1—O1—C1	−48.4 (2)	O3—Sm1—N4—O8	−142.62 (17)
O11—Sm1—O1—C1	−100.3 (2)	O2—Sm1—N4—O8	169.89 (15)
O6—Sm1—O1—C1	107.4 (2)	O9—Sm1—N4—O8	−179.2 (3)
O8—Sm1—O1—C1	1.8 (2)	O5—Sm1—N4—O8	−8.53 (18)
O4—Sm1—O1—C1	93.8 (2)	O12—Sm1—N4—O8	83.81 (19)
N5—Sm1—O1—C1	−75.5 (2)	O11—Sm1—N4—O8	131.28 (19)
N3—Sm1—O1—C1	80.9 (2)	O6—Sm1—N4—O8	−47.9 (2)
O3—Cu1—O2—C7	−168.4 (2)	O1—Sm1—N4—O8	57.26 (19)
N1—Cu1—O2—C7	−2.0 (2)	O4—Sm1—N4—O8	−87.65 (17)
N2—Cu1—O2—C7	139.3 (3)	N5—Sm1—N4—O8	108.0 (2)
Sm1—Cu1—O2—C7	178.4 (2)	N3—Sm1—N4—O8	−24.14 (19)
O3—Cu1—O2—Sm1	13.15 (8)	O3—Sm1—N4—O9	36.59 (18)
N1—Cu1—O2—Sm1	179.54 (8)	O2—Sm1—N4—O9	−10.9 (3)
N2—Cu1—O2—Sm1	−39.1 (3)	O5—Sm1—N4—O9	170.68 (17)
O3—Sm1—O2—C7	169.5 (2)	O12—Sm1—N4—O9	−96.99 (19)
O9—Sm1—O2—C7	−140.60 (17)	O11—Sm1—N4—O9	−49.52 (19)
O5—Sm1—O2—C7	43.43 (19)	O6—Sm1—N4—O9	131.30 (17)
O12—Sm1—O2—C7	−58.64 (19)	O8—Sm1—N4—O9	179.2 (3)
O11—Sm1—O2—C7	−92.6 (2)	O1—Sm1—N4—O9	−123.54 (17)
O6—Sm1—O2—C7	74.46 (18)	O4—Sm1—N4—O9	91.56 (18)
O1—Sm1—O2—C7	−4.31 (16)	N5—Sm1—N4—O9	−72.8 (2)
O4—Sm1—O2—C7	135.11 (17)	N3—Sm1—N4—O9	155.07 (17)
N5—Sm1—O2—C7	−74.5 (2)	Sm1—O11—N5—O12	5.2 (4)
N3—Sm1—O2—C7	58.02 (18)	Sm1—O11—N5—O13'	169 (2)
O3—Sm1—O2—Cu1	−11.98 (7)	Sm1—O11—N5—O13	−152 (3)
O9—Sm1—O2—Cu1	37.90 (12)	Sm1—O12—N5—O11	−5.2 (4)
O5—Sm1—O2—Cu1	−138.07 (8)	Sm1—O12—N5—O13'	−164 (4)
O12—Sm1—O2—Cu1	119.87 (10)	Sm1—O12—N5—O13	158.8 (16)
O11—Sm1—O2—Cu1	85.86 (13)	O3—Sm1—N5—O11	14.7 (3)
O6—Sm1—O2—Cu1	−107.04 (9)	O2—Sm1—N5—O11	−42.9 (3)
O1—Sm1—O2—Cu1	174.19 (10)	O9—Sm1—N5—O11	86.4 (3)
O4—Sm1—O2—Cu1	−46.39 (10)	O5—Sm1—N5—O11	−159.9 (3)
N5—Sm1—O2—Cu1	104.03 (14)	O12—Sm1—N5—O11	174.6 (4)
N3—Sm1—O2—Cu1	−123.47 (9)	O6—Sm1—N5—O11	−100.3 (4)
O2—Cu1—O3—C19	160.8 (2)	O8—Sm1—N5—O11	135.7 (3)
N1—Cu1—O3—C19	102.2 (3)	O1—Sm1—N5—O11	−104.9 (3)
N2—Cu1—O3—C19	−32.3 (2)	O4—Sm1—N5—O11	86.1 (3)
Sm1—Cu1—O3—C19	174.6 (3)	N3—Sm1—N5—O11	−135.2 (3)
O2—Cu1—O3—Sm1	−13.84 (8)	O3—Sm1—N5—O12	−159.9 (2)
N1—Cu1—O3—Sm1	−72.4 (3)	O2—Sm1—N5—O12	142.5 (2)
N2—Cu1—O3—Sm1	153.08 (10)	O9—Sm1—N5—O12	−88.2 (2)
O2—Sm1—O3—C19	−162.4 (2)	O5—Sm1—N5—O12	25.6 (3)
O9—Sm1—O3—C19	56.63 (19)	O11—Sm1—N5—O12	−174.6 (4)
O5—Sm1—O3—C19	−62.6 (2)	O6—Sm1—N5—O12	85.1 (2)
O12—Sm1—O3—C19	112.1 (2)	O8—Sm1—N5—O12	−38.9 (2)
O11—Sm1—O3—C19	130.3 (2)	O1—Sm1—N5—O12	80.5 (2)
O6—Sm1—O3—C19	−88.4 (2)	O4—Sm1—N5—O12	−88.5 (3)
O8—Sm1—O3—C19	24.2 (2)	N3—Sm1—N5—O12	50.2 (2)
O1—Sm1—O3—C19	−155.86 (18)	O3—Sm1—N5—O13'	−23 (2)
O4—Sm1—O3—C19	−17.83 (18)	O2—Sm1—N5—O13'	−81 (2)
N5—Sm1—O3—C19	124.0 (2)	O9—Sm1—N5—O13'	49 (2)
N3—Sm1—O3—C19	−79.2 (2)	O5—Sm1—N5—O13'	162 (2)
O2—Sm1—O3—Cu1	12.22 (7)	O12—Sm1—N5—O13'	137 (2)
O9—Sm1—O3—Cu1	−128.73 (10)	O11—Sm1—N5—O13'	−38 (2)
O5—Sm1—O3—Cu1	112.07 (10)	O6—Sm1—N5—O13'	−138 (2)
O12—Sm1—O3—Cu1	−73.23 (13)	O8—Sm1—N5—O13'	98 (2)
O11—Sm1—O3—Cu1	−55.11 (11)	O1—Sm1—N5—O13'	−143 (2)
O6—Sm1—O3—Cu1	86.26 (10)	O4—Sm1—N5—O13'	48 (2)
O8—Sm1—O3—Cu1	−161.16 (8)	N3—Sm1—N5—O13'	−173 (2)
O1—Sm1—O3—Cu1	18.78 (12)	O3—Sm1—N5—O13	148.4 (16)
O4—Sm1—O3—Cu1	156.81 (11)	O2—Sm1—N5—O13	90.8 (16)
N5—Sm1—O3—Cu1	−61.33 (11)	O9—Sm1—N5—O13	−140.0 (16)
N3—Sm1—O3—Cu1	95.42 (10)	O5—Sm1—N5—O13	−26.2 (16)
O3—Sm1—O4—C18	16.38 (17)	O12—Sm1—N5—O13	−51.7 (16)
O2—Sm1—O4—C18	50.38 (19)	O11—Sm1—N5—O13	133.7 (16)
O9—Sm1—O4—C18	−71.38 (18)	O6—Sm1—N5—O13	33.4 (16)
O5—Sm1—O4—C18	164.60 (18)	O8—Sm1—N5—O13	−90.6 (16)
O12—Sm1—O4—C18	−110.1 (2)	O1—Sm1—N5—O13	28.8 (16)
O11—Sm1—O4—C18	−33.3 (2)	O4—Sm1—N5—O13	−140.2 (16)
O6—Sm1—O4—C18	112.84 (18)	N3—Sm1—N5—O13	−1.5 (16)
O8—Sm1—O4—C18	−125.32 (19)	C1—O1—C2—C3	−8.8 (4)
O1—Sm1—O4—C18	126.56 (17)	Sm1—O1—C2—C3	174.2 (2)
N5—Sm1—O4—C18	−71.2 (3)	C1—O1—C2—C7	171.3 (2)
N3—Sm1—O4—C18	138.85 (19)	Sm1—O1—C2—C7	−5.6 (3)
O3—Sm1—O4—C20	−170.0 (3)	O1—C2—C3—C4	−179.8 (2)
O2—Sm1—O4—C20	−136.0 (3)	C7—C2—C3—C4	0.0 (4)
O9—Sm1—O4—C20	102.2 (3)	C2—C3—C4—C5	−1.3 (4)
O5—Sm1—O4—C20	−21.8 (3)	C3—C4—C5—C6	0.8 (4)
O12—Sm1—O4—C20	63.5 (3)	C4—C5—C6—C7	1.0 (4)
O11—Sm1—O4—C20	140.3 (3)	C4—C5—C6—C8	−178.0 (2)
O6—Sm1—O4—C20	−73.6 (3)	Cu1—O2—C7—C6	5.3 (3)
O8—Sm1—O4—C20	48.3 (3)	Sm1—O2—C7—C6	−176.54 (16)
O1—Sm1—O4—C20	−59.8 (3)	Cu1—O2—C7—C2	−174.92 (16)
N5—Sm1—O4—C20	102.4 (3)	Sm1—O2—C7—C2	3.2 (3)
N3—Sm1—O4—C20	−47.6 (3)	C5—C6—C7—O2	177.5 (2)
O3—Sm1—O5—N3	−38.23 (19)	C8—C6—C7—O2	−3.5 (4)
O2—Sm1—O5—N3	35.50 (18)	C5—C6—C7—C2	−2.3 (3)
O9—Sm1—O5—N3	−140.79 (16)	C8—C6—C7—C2	176.7 (2)
O12—Sm1—O5—N3	145.57 (17)	C3—C2—C7—O2	−178.1 (2)
O11—Sm1—O5—N3	121.73 (18)	O1—C2—C7—O2	1.8 (3)
O6—Sm1—O5—N3	−4.23 (15)	C3—C2—C7—C6	1.7 (4)
O8—Sm1—O5—N3	−149.23 (18)	O1—C2—C7—C6	−178.4 (2)
O1—Sm1—O5—N3	79.68 (16)	C9—N1—C8—C6	−176.2 (2)
O4—Sm1—O5—N3	−77.45 (17)	Cu1—N1—C8—C6	5.7 (4)
N5—Sm1—O5—N3	134.86 (17)	C7—C6—C8—N1	−2.4 (4)
O3—Sm1—O6—N3	159.81 (17)	C5—C6—C8—N1	176.6 (2)
O2—Sm1—O6—N3	−139.04 (17)	C8—N1—C9—C10	98.8 (3)
O9—Sm1—O6—N3	91.2 (2)	Cu1—N1—C9—C10	−83.0 (3)
O5—Sm1—O6—N3	4.26 (15)	N1—C9—C10—C11	60.0 (3)
O12—Sm1—O6—N3	−41.0 (2)	C9—C10—C11—C12	48.2 (4)
O11—Sm1—O6—N3	−119.9 (2)	C13—N2—C12—C11	−164.1 (3)
O8—Sm1—O6—N3	39.08 (18)	Cu1—N2—C12—C11	14.4 (4)
O1—Sm1—O6—N3	−73.74 (16)	C10—C11—C12—N2	−90.6 (3)
O4—Sm1—O6—N3	97.79 (17)	C12—N2—C13—C14	173.8 (3)
N5—Sm1—O6—N3	−78.3 (3)	Cu1—N2—C13—C14	−4.7 (4)
O3—Sm1—O8—N4	43.42 (19)	N2—C13—C14—C19	−13.2 (5)
O9—Sm1—O8—N4	0.44 (16)	N2—C13—C14—C15	171.3 (3)
O5—Sm1—O8—N4	170.83 (19)	C19—C14—C15—C16	2.1 (4)
O12—Sm1—O8—N4	−86.22 (18)	C13—C14—C15—C16	177.7 (3)
O11—Sm1—O8—N4	−51.3 (2)	C14—C15—C16—C17	−1.2 (5)
O6—Sm1—O8—N4	142.29 (16)	C15—C16—C17—C18	−0.8 (5)
O1—Sm1—O8—N4	−136.52 (16)	C16—C17—C18—O4	−178.0 (3)
O4—Sm1—O8—N4	82.69 (17)	C16—C17—C18—C19	2.0 (4)
N5—Sm1—O8—N4	−69.3 (2)	C20—O4—C18—C17	−8.9 (4)
N3—Sm1—O8—N4	158.04 (17)	Sm1—O4—C18—C17	165.2 (2)
O3—Sm1—O9—N4	−143.42 (18)	C20—O4—C18—C19	171.1 (3)
O2—Sm1—O9—N4	173.54 (15)	Sm1—O4—C18—C19	−14.7 (3)
O5—Sm1—O9—N4	−10.6 (2)	Cu1—O3—C19—C14	25.2 (4)
O12—Sm1—O9—N4	74.48 (19)	Sm1—O3—C19—C14	−161.0 (2)
O11—Sm1—O9—N4	127.4 (2)	Cu1—O3—C19—C18	−156.08 (19)
O6—Sm1—O9—N4	−72.5 (2)	Sm1—O3—C19—C18	17.8 (3)
O8—Sm1—O9—N4	−0.44 (15)	C15—C14—C19—O3	177.9 (3)
O1—Sm1—O9—N4	83.5 (2)	C13—C14—C19—O3	2.5 (4)
O4—Sm1—O9—N4	−79.10 (17)	C15—C14—C19—C18	−0.9 (4)
N5—Sm1—O9—N4	101.0 (2)	C13—C14—C19—C18	−176.3 (3)
N3—Sm1—O9—N4	−33.9 (2)	C17—C18—C19—O3	−180.0 (2)
O3—Sm1—O11—N5	−166.3 (3)	O4—C18—C19—O3	0.0 (4)
O2—Sm1—O11—N5	133.1 (3)	C17—C18—C19—C14	−1.2 (4)
O9—Sm1—O11—N5	−85.9 (3)	O4—C18—C19—C14	178.8 (2)
